# Computational and Bioinformatics Frameworks for Next-Generation Whole Exome and Genome Sequencing

**DOI:** 10.1155/2013/730210

**Published:** 2013-01-13

**Authors:** Marisa P. Dolled-Filhart, Michael Lee, Chih-wen Ou-yang, Rajini Rani Haraksingh, Jimmy Cheng-Ho Lin

**Affiliations:** Rare Genomics Institute, 4100 Forest Park Avenue, Suite 204, St. Louis, MO 63108, USA

## Abstract

It has become increasingly apparent that one of the major hurdles in the genomic age will be the bioinformatics challenges of next-generation sequencing. We provide an overview of a general framework of bioinformatics analysis. For each of the three stages of (1) alignment, (2) variant calling, and (3) filtering and annotation, we describe the analysis required and survey the different software packages that are used. Furthermore, we discuss possible future developments as data sources grow and highlight opportunities for new bioinformatics tools to be developed.

## 1. Introduction

Without doubt, the development of next-generation sequencing has transformed biomedical research. Multiple second generation sequencing platforms, such as Roche/454, Illumina/Solexa, AB/SOLiD, and LIFE/Ion Torrent, have made high-throughput genetic analysis more readily accessible to researchers and even clinicians [1]. On the horizon, third generation sequencing technologies, such as Oxford Nanopore, Genia, NABsys, and GnuBio, will continue to increase throughput capabilities and decrease the cost of sequencing. With each new generation of sequencing technology, there is an exponential increase in the flood of data. The true challenges of high throughput sequencing will be bioinformatics. As ever larger datasets become more affordable, computational analysis rather than sequencing will be the rate-limiting factor in genomics research. In this paper, we provide an overview of the current computational framework and options for genomic analysis and provide some outlook on future developments and upcoming needs.

**Figure 1:**
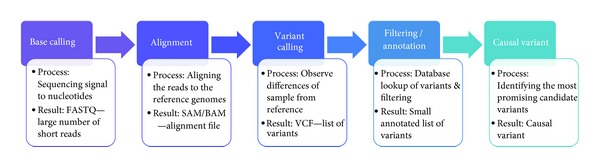
Next-generation sequencing bioinformatics workflow.

In this paper, we will discuss some of the options in each of the steps and provide a global outlook on the software “pipelines” currently in development (Figure 1).

## 2. Overview

While different sequencing technologies may use different initial raw data (e.g., imaging files or fluctuations in current), the eventual outputs are nucleotide base calls. Short strings of these bases, varying from dozens to hundreds of base pairs for each fragment, are combined together, often in a form of a FASTQ file. From here, bioinformatics analysis of the sequence falls into three general steps: (1) alignment, (2) variant calling, (3) filtering and annotation.

The first step is alignment—matching each of the short reads to positions on a reference genome (for the purposes of this paper, the human genome). The resulting sequence alignment is stored in a SAM (sequence alignment/map) or BAM (binary alignment/map) file [2]. The second step is variant calling—comparing the aligned sequences with known sequences to determine which positions deviate from the reference position. The produces a list of positions or calls recorded in a VCF (variant call format) file [3]. The third step includes both filtering as well as annotation. Filtering takes the tens of thousands of variants and reduces them to a smaller set. For cancers, this involves comparing cancerous cell genomes to normal genomes. For family data, it involves selecting variants that conform to a specific genetic inheritance pattern. Annotation involves querying known information about each variant that is detected. Annotation may reveal, for example, that a variant is an already-known single nucleotide polymorphism, that a functional effect has already been predicted, that the function or activity of the gene in question is already known, or even that an associated disease has been identified.

Ultimately, the optimal result from the analysis is a small number of well-annotated variants that can explain a biological phenomenon. For example, for a Mendelian disease, analysis could identify the causative variant or gene. For cancer, analysis may point to driver mutations or targetable genes. Starting from base calls and ending with biologically important genetic variants, each step of analysis may be performed using one of many pieces of software. This paper discusses several of the bioinformatics options for each of these three steps.

## 3. Alignment

Alignment is the process of mapping short nucleotide reads to a reference genome. Because each of the millions of short reads must be compared to the 3 billion possible positions within the human genome, this computational step is not trivial. Software must assess the likely starting point of each read within the reference genome, and the task is complicated by the volume of short reads, unique versus non-unique mapping, and variation in base quality. This step is thus computationally intense and time consuming [4]. It is also a critical step, as any errors in alignment to the reference genome will be carried through to the rest of the analysis.

The Sequence Alignment/Map (SAM) and Binary Alignment/Map (BAM) formats are the standard file formats for storing NGS read alignments [2]. There are various software programs, some commercially available and others freely available to the scientific community, that can be used to perform sequencing read alignment. Various programs differ in speed and accuracy. Most alignment algorithms use an indexing method in order to more rapidly narrow down potential alignment locations within the reference genome with ungapped alignment, although other algorithms allow for gapped alignment. Different approaches to alignment involve hash tables, spaced seeds, and/or contiguous seeds. This method also enables comparison of differing output structures (single versus multiple possible alignment outputs) [5].

Short reads generated from NGS may either be single-end reads or paired-end reads from the sample, and may range from dozens to hundreds of base pairs [5]; these reads need to be aligned correctly to their appropriate location within the reference genome. Algorithms typically utilize a hash-based index (e.g., MAQ, ELAND), BWT-based index (e.g., BWA, Bowtie, SOAP2), genome-based hash (e.g., Novoalign, SOAP), or a spaced-seed approach (e.g., SHRiMP). Some algorithms report the “best” match using heuristic approaches (e.g., BWA, Bowtie, MAQ), while others allow for all possible matches (e.g., SOAP3, SHRiMP). Algorithms differ in whether they can handle both single-end and paired-end reads, or just one type (e.g., SARUMAN for single-end reads), and whether they can perform gapped alignment (e.g., BWA, Bowtie2) in addition to ungapped alignment (e.g., MAQ, Bowtie). Some algorithms focus on speed (e.g., BWA, Bowtie), some on sensitivity (e.g., Novoalign), and some algorithms aim to the two (e.g., Stampy).  Table 1 provides a listing of relevant algorithms for alignment of short reads to the reference genome. While there has been previous comparisons about these algorithms [6], we describe some of the newer programs, such as Bowtie and Bowtie 2, or SOAP/SOAP2/SOAP3, and others below.

### 3.1. Bowtie/Bowtie 2

The Bowtie algorithm is both ultrafast and memory efficient [7] due to its use of a refinement of the FM Index, which itself utilizes the Burrows-Wheeler transformation for ultrafast and memory-efficient alignment of reads to a reference genome. Bowtie improves upon BWT with a novel quality-aware backtracking algorithm that permits mismatches. However, there may be some tradeoffs between speed and alignment quality using this algorithm [5]. Bowtie2 allows for analysis of gapped reads, which may result either from true insertions or deletions, or from sequencing errors. The newer adaptions utilize full-text minute indices and hardware-accelerated dynamic programming algorithms to optimize both speed and accuracy [8].

### 3.2. BWA/BWA-SW

The BWA approach, based on BWT, provides efficient alignment of short reads against the reference genome [9]. This is the most commonly used approach for sequence alignment, and followed the development of the first-generation hash-table based alignment algorithm MAQ [10]. BWA improved upon MAQ by allowing for gapped alignment of single end reads, which is important for longer reads that may contain indels, and allowed for increased speed. BWA-SW allows for matches without heuristics and alignment of longer sequences [11].

### 3.3. mrFAST/mrsFAST

In contrast to algorithms focused on “unique” alignment of regions of the genome and selection of the “best” match, mrFAST [12] and mrsFAST [13] allow for rapid assessment of copy-number variation and assignment of sequences into both unique and the more complex duplicated regions of the genome [14, 15]. The methodology of these algorithms is a seed-and-extend approach similar to BLAST, which uses hash tables to index the reference genome. These algorithms can handle smaller structural variants (e.g., indels) and larger structural variants such as insertions, deletions, inversions, CNVs, and segmental duplications in a cache-oblivious manner.

### 3.4. SHRiMP/SHRiMP2

Developed to handle a greater number of polymorphisms by utilizing a statistical model to screen out false positive hits, SHRiMP [16] can be utilized for color-spaced reads from AB SOLiD sequencers and can also be used for regular letter-space reads. SHRiMP2 [17] enables direct alignment for paired-reads and uses multiple spaced seeds, but instead of using indexed reads like SHRiMP, SHRiMP2 switched to an indexing method like Bowtie and BWA.

### 3.5. SOAP/SOAPv2/SOAPv3

SOAP was developed for use in gapped and ungapped alignment of short reads using a seed strategy for either single-read or pair-end reads, and can also be applied to small RNA and mRNA tag sequences [18]. SOAP2 reduced memory usage and increased speed using BWT for hash-based indexing instead of the seed algorithm, and also includes SNP detection [19]. SOAP3 is a GPU (graphics processing unit) version of the compressed full-text index-based SOAP2, which allows for a speed improvement [20].

## 4. Variant Calling

After alignment of the short reads to the reference genome, the next step in the bioinformatics process is variant calling. Since the short reads are already aligned, the sample genome can be compared to the reference genome and variants can then be identified. These variants may be responsible for disease, or they may simply be genomic noise without any functional effect. Variant call format (VCF) is the standardized generic format for storing sequence variation including SNPs, indels, larger structural variants and annotations [3]. The computational challenges in SNP (variant) calling are due to the issues in identifying “true” variants versus alignment and/or sequencing errors. Yet the ability to detect SNPs with both high sensitivity and specificity is a key step in identifying sequence variants associated with disease, detection of rare variants, and assessment of allele frequencies in populations.

The difficulty of variant calling is complicated by three factors: (1) the presence of indels, which represent a major source of false positive SNV identifications, especially if alignment algorithms do not perform gapped alignments; (2) errors from library preparation due to PCR artifacts and variable GC content in the short reads unless paired-end sequencing is utilized; and (3) variable quality scores, with higher error rates generally found at bases at the ends of reads [4]. Therefore, the rate of false positive and false negative calls of SNVs and indels is a concern. A detailed review of SNP-calling algorithms and challenges recommends recalibration of per-base quality scores (e.g., GATK, SOAPsnp), use of an alignment algorithm with high sensitivity (e.g., Novoalign, Stampy), and SNP calling using Bayesian procedures or likelihood ratio tests and incorporation of linkage disequilibrium to improve SNP call accuracy [21]. We provide an overview of some of the software packages for variant calling below.

### 4.1. The Genome Analysis ToolKit (GATK)

Developed by the Broad Institute, the Genome Analysis ToolKit (GATK) is one of the most popular methods for variant calling using aligned reads. It is designed in a modular way and is based on the MapReduce functional programming approach [22]. The package has been used for projects such as The Cancer Genome Atlas [23] and the 1000 Genomes Project [24] that have covered analyses of HLA typing, multiple-sequence realignment, quality score recalibration, multiple-sample SNP genotyping and indel discovery and genotyping [22].

### 4.2. SOAPsnp

Developed by the Beijing Genome Institute, SOAPsnp is an open source algorithm (http://soap.genomics.org.cn/) that requires access to a high-quality variant database using SOAP alignment results as an input [18]. It can be used for consensus calling and SNP detection for the Illumina Genome Analyzer platform and utilizes the phred-like quality score to calculate the likelihood of each genotype based on the alignment results and sequencing quality scores. Building upon the speed of the alignment algorithm Bowtie [7] and using SOAPsnp for SNP calling, an open source cloud-computing tool called Crossbow [7] was developed to perform both alignment and SNP calling.

### 4.3. VarScan/VarScan2

Developed by the Genome Institute at Washington University in St. Louis, VarScan (http://genome.wustl.edu/tools/cancer-genomics/) is an open source tool for short read variant detection of SNPs and indels that is compatible with multiple sequencing platforms and aligner algorithms such as Bowtie and Novoalign [25]. It can detect variants at 1% frequency, which can be useful for pooled samples; VarScan permits analysis of individual samples as well. VarScan2 [26] includes some improvements upon VarScan, such as the ability to analysis tumor-normal sample pairs for somatic mutations, LOH (loss of heterozygosity) and CNAs (copy number alterations). This program reads tumor and normal sample Samtools pileup or mpileup output simultaneously for pairwise comparisons of base calling and normalized sequence depth at each position.

### 4.4. ATLAS 2

Developed by the Baylor Genome Center, Atlas 2 can be used for variant calling of aligned data from multiple NGS platforms on a range of computing platforms [27]. Atlas 2 can also be implemented via a web resource called Genboree Workbench (http://www.genboree.org/). A few other web-based analysis tools are available such as DNANexus (http://www.dnanexus.com/) and Galaxy [28]. Details of Atlas 2 in comparison to other variant calling algorithms such as SAMtools mpileup and GATK are included in Challis et al. [27], and reviewed by Ji [29].

## 5. Insertions and Deletions

While the majority of research has focused on diseases associated with SNPs, indel (insertion and deletion) mutations are a common polymorphism that can also demonstrate to biological effects. Studies have shown that small indels might be highly associated with neuropsychiatric diseases such as schizophrenia, autism, mental retardation, and Alzheimer's disease [30].

In addition, the presence of certain indels is associated with the disease progression of HBV-induced hepatocellular carcinoma (HCC) in the Korean population [31]. Indels are also used as genetic markers in natural populations [32]. With the advance of sequencing platforms and analysis tools, detection of indels through NGS has become more common. However, accurate mapping of indels to the reference genome is challenging, because it requires approaches that involve complicated gapped alignment and paired-end sequence inference [9]. Moreover, the occurrence rate of indels is approximately 8-fold lower than that of SNPs [33]. An optimal combination of both alignment and indel-calling algorithms is essential for identifying indels with high sensitivity and specificity. One review evaluated the performance of various alignment tools on microindel detection, and recommended single-end reads gapped alignment mapping tools such as BWA and Novoalign [34]. Various software approaches have been developed to identify indels, including a pattern growth approach (e.g., Pindel) and a Bayesian procedure (e.g., Dindel). A detailed review by Neuman et al. evaluated the performance of several difference indel-calling programs in the presence of varying parameters (read depth, read length, indel size, and frequency). By using both simulated and real data that included the *Caenorhabditis elegans *genome, they observed that Dindel has the highest sensitivity (indels found) at low coverage, although Dindel is only suitable for Illumina data analysis. VarScan and GATK require additional parameter adjustments, such as high coverage for VarScan, to reach their best performance. This review provides information for appropriate tool selection and parameter optimization to assist successful experimental designs and recommends Dindel as a suitable tool for low coverage experiments. Below, we survey the tools that have been commonly used for indel calling.

### 5.1. Pindel

Pindel is a software program which implements a pattern growth approach to detect breakpoints of large deletions (1–10 kb) and medium sized insertions (1 bp–20 bp) from paired-end short reads in NGS data [35]. A recent, more advanced, version, Pindel2, has been introduced which includes the ability to identify insertions of any size, inversions and tandem duplications [35]. Pindel has been used for the 1000 Genomes Project (http://www.1000genomes.org/) [36], the Genome of the Netherlands project, and the Cancer Genome Atlas [23].

### 5.2. Dindel

Developed by the Welcome Trust Sanger Institute, Dindel is an open-source program that utilizes a Bayesian approach for calling small (<50 bp) insertions and deletions (http://www.sanger.ac.uk/resources/software/dindel/) [37]. Principally, this algorithm realigns sequence reads mapped to a variety of candidate haplotypes that represent alternative sequences to the reference. Dindel has been used in the 1000 Genomes Project call sets and can only analyze data from Illumina.

### 5.3. GATK

As described in the variant calling section, the Genome Analysis ToolKit (GATK), which provides a collection of data analysis tools, can also allow indel calling based on the MapReduce programming approach [22]. Details of GATK in comparison to other indel calling methods including Dindel (VarScan, SAMtools mpileup) are evaluated in Neuman et al. [38].

## 6. Filtering and Annotation

After alignment and variant calling, a list of thousands of potential differences between the genome under study and the reference genome is generated. The next step is to determine which of these variants are likely to contribute to the pathological process under study. The third step involves a combination of both filtering (removing variants that fit specific genetic models or are not present in normal tissue) as well as annotation (looking up information about variants and identifying ones that fit the biological process).

Filtering can be done with a genetic pedigree or with cancer and normal samples from the same individual. In the instance of cancer, a common method is removing variants that are present in both the cancer sample and the normal sample, leaving only somatic variants, which have mutated from the germline sequence. In the instance of a pedigree, filtering can be done based on the different inheritance patterns. For example, if the inheritance pattern is autosomal recessive, the variants that are heterozygous in the parents and homozygous in the child can be chosen. Similar methods can be done with larger pedigrees based on the inheritance pattern.

In addition to filtering, further selection of causal variants can be based on existing annotation or predicted functional effect. Many tools exist to examine relevant variants by referencing previously known information about their biological functions and inferring potential effects based on their genomic context. In addition, many tools have been developed to identify genetic variants that cause disease pathogenesis or phenotypic variance [39]. Rare nonsynonymous SNPs are SNPs that cause amino acid substitution (AAS) in the coding region, which potentially affect the function of the protein coded and could contribute to disease.

The advance of exome and genomic sequencing is yielding an extensive number of human genetic variants, and a number of disease-associated SNVs can be identified following alignment and variant calling. Unlike nonsense and frameshift mutations, which often result in a loss of protein function, pinpointing disease-causal variants among numerous SNVs has become one of the major challenges due to the lack of genetic information. For instance, ~1,300 loci are shown to be associated with ~200 diseases by GWASs but only a few of these loci have been identified as disease-causing variants [40]. Exome sequencing enables the identification of more novel genetic variants than previously possible, but it still requires computational and experimental approaches to predict whether a variant is deleterious. To this end, several approaches have been developed to identify rare nonsynonymous SNPs that cause amino acid substitution (AAS) in the coding region. The major principle of the protein-sequence-based methods to predict deleteriousness in the coding sequence is based on comparative genomics and functional genomics. Comparative sequencing analysis assumes that amino acid residues that are critical for protein function should be conserved among species and homologous proteins; therefore, mutations in highly conserved sites are more likely to result in more deleterious effect. Other modalities to predict disease-causing variants include protein biochemistry, such as amino acid charge, the presence of a binding site, and structure information of protein. SNVs that are predicted to alter protein feature (such as polarity and hydropathy) and structure (binding ability and alteration of secondary/tertiary structure) have a higher probability of being deleterious.

Although the majority of research has focused on protein-altering variants, noncoding variants constitute a large portion of human genetic variation. Results obtained from GWAS indicate that ~88% of trait-associated weak effect variants are found in noncoding regions, demonstrating the importance of functional annotation of both coding and noncoding variants [41]. Computational tools for protein-sequence-based prediction of deleteriousness fall into two categories: constraint-based predictors such as MAPP and SIFT, and trained classifiers such as MutationTaster and polyPhen. In addition to protein-sequence-based methods, another way to prioritize disease-casual SNVs is through nucleotide-sequence-based prediction in noncoding and coding DNA. This process also utilizes comparative genomics to predict deleteriousness, and is used by programs such as phastCons, GERP, and Gumby. In one detailed review of disease-causing variant identification, the authors introduced the concepts and tools that allow genetic annotation of both coding and noncoding variants [39]. They also compared the relative utility of nucleotide- and protein-based approaches using exome data, finding that nucleotide-based constraint scores defined by Genomic Evolutionary Rate Profiling (GERP) and protein-based deleterious impact scores provided by PolyPhen were similar for two Mendelian diseases, suggesting that nucleotide-based prediction can be as powerful as protein-based metrics [39]. Below, we survey tools that are helpful identifying disease-causal variants among numerous candidates.

### 6.1. Sorting Intolerant from Tolerant (SIFT)

Sorting Intolerant From Tolerant (SIFT) (http://sift.jcvi.org/) prediction is based on conserved amino acid residues through different species using comparative sequencing analysis through PSI-BLAST [42]. This relies on the presumption that amino acid residues that are essential for protein function should be evolutionally conserved by natural selection. Therefore, SNPs resulting in AAS on the conservative residues are more likely to be deleterious.

### 6.2. PolyPhen

PolyPhen/PolyPhen2 (http://genetics.bwh.harvard.edu/pph2/) algorithm predicts the potential impact of AAS on the structure and function of human protein based on protein sequence, phylogenetic and structural information [43]. An amino acid replacement might occur at a specific site where binding to other molecules or the formation of a secondary/tertiary structure is disrupted. Therefore, PolyPhen determines if the AAS is found at a site which is annotated as a disulfide bond, an active site, a binding site, or a specific motif such as transmembrane domain. Another function of PolyPhen is to compare the sequence and polymorphic regions of homologous proteins in the same family to identify AASs that are rare or never observed in the family. In addition, PolyPhen also maps of the substitution site to the known 3-dimensional protein structure to assess if an AAS has the potential to destroy protein structure via an alteration of, for example, the hydrophobic core of a protein, electrostatic interactions, or interactions with ligands or other molecules.

### 6.3. VariBench

VariBench (http://structure.bmc.lu.se/VariBench/) is the first benchmark database that provides testing and training tools for computational variation effect prediction [44]. It comprises experimentally validated variation datasets collected from the literature and relevant databases. The datasets housed in VariBench enable identification of variants that affect protein tolerance, protein stability, transcription factor binding sites, and splice sites. Additionally, VariBench maps variant positions to the DNA, RNA, and protein sequences at RefSeq, and to the 3-dimensional protein structures at Protein Data Bank (PDB).

### 6.4. snpEFF

snpEFF is an open source, Java-based program that rapidly categorizes SNP, indel, and MNP variants in genomic sequences as having either high, medium, low or modifier functional effects [45]. Variant annotation is based on genomic location (intron, exon, untranslated region, upstream, downstream, splice site, intergenic region) and predicted coding effect (synonymous/nonsynonymous amino acid replacement, gain/loss of start/stop site, frameshift mutations). The program may find several different functions for a single variant due to competing predictions based on alternative transcripts. snpEFF uses a VCF input and output style. Currently snpEFF does not support structural variants but there are plans to incorporate such support soon. snpEFF is compatible with GATK and Galaxy, which are popular variant-calling toolkits. The program currently supports 260 genome versions and can be used with custom genomes and annotations.

### 6.5. The SNPeffect Database

The SNPeffect Annotation database (http://snpeffect.switchlab.org/) uses sequence and structure information to predict the effect of protein-coding SNVs on the structural phenotype of proteins [46]. It is primarily focused on disease-causing and polymorphic variants in the human proteome. This program compares variant protein predictions to wild type protein information from the UniProtKB database, which currently contains more than 60,000 variant proteins. Variant characterization is achieved by integrating aggregation, amyloid prediction, chaperone-binding prediction, and protein stability analysis information by applying several algorithms to each wild type and mutant protein. The first algorithm, TANGO, detects regions that are prone to aggregation and calculates a score difference between the mutant and wild type protein. The WALTZ algorithm is applied to predict amyloid-forming regions in protein sequences using a position-specific scoring matrix to deduce amyloid-forming propensity. LIMBO is an algorithm that predicts chaperone binding sites for the Hsp70 chaperones. In cases where structural information is available, the FoldX algorithm is used to calculate the difference in free energy between the mutated protein and the wild type and determine whether the mutation stabilizes or destabilizes the structure. Mutations are also characterized as falling into catalytic sites according to information in the Catalytic Site Atlas or not, and falling into known domains or not. Subcellular information is predicted using PSORT.

### 6.6. SeattleSeq

SeattleSeq (http://snp.gs.washington.edu/SeattleSeqAnnotation/) annotates known and novel SNPs with biological functions, protein positions and amino-acid changes, conservation scores, HapMap frequencies, PolyPhen predictions, and clinical associations based on an integrated database. Most of the annotation information is derived from the Genome Variation Server (http://gvs.gs.washington.edu/GVS134/), which includes information from dbSNP as well as other sources. The algorithm accepts input files in a number of formats including GATK and VCF output styles. Currently, annotation of indels is limited.

### 6.7. ANNOVAR

The ANNOVAR software tool (http://www.openbioinformatics.org/annovar/) utilizes up-to date information to rapidly functionally annotate genetic variants called from sequencing data [47]. ANNOVAR works on a number of diverse genomes including hg18, hg19, mouse, worm, fly, and yeast. The annotation system allows the user flexibility in the set of genomic regions that are queried. Annotations can be gene-based (users can select the gene definition system; RefSeq, UCSC, ENSEMBL, GENCODE, etc.), region-based (transcription factor binding sites, DNAse I hypersensitivity sites, ENCODEmethylation sites, segmental duplication sites, DGV sites, etc.), filter-based (e.g., using only variants reported in dbSNP, or only variants with MAF > 1%), or based on any of many other user-driven functionalities.

### 6.8. The Variant Annotation, Analysis and Search Tool (VAAST)

The Variant Annotation, Analysis and Search Tool (VAAST) identifies damaged genes and deleterious variants in personal genome sequences using a probabilistic search method [48]. The tool utilizes both existing amino acid substitution and aggregative approaches to variant prioritization and combines them into a single unified likelihood-framework. This method increases the accuracy with which disease causing variants are identified. VAAST scores both coding and noncoding, and both rare and common, variants simultaneously and aggregates this information to identify disease causing variants.

### 6.9. The Variant Analysis Tool (VAT)

The Variant Analysis Tool, VAT, (http://vat.gersteinlab.org/) functionally annotates variants called from personal genomes at the transcript level and provides summary statistics across genes and individuals [49]. VAT is a computational framework that can be implemented through a command-line interface, a web application, or a virtual machine in a cloud-computing environment. This tool has been utilized extensively to annotate loss-of-function variants obtained as part of the 1000 Genomes Project [50]. The VAT modules *snpMapper*, *indelMapper and svMapper* relate SNPs, indels and SVs to protein-coding genes while the *genericMapper* module relates variants to noncoding regions of the genome. Transcript-level analysis allows identification of affected isoforms. VAT outputs VCF files as well as visualization summarizing the biological impact of annotated variants.

### 6.10. VARIANT

VARIANT (VARIant ANalysis Tool) (http://variant.bioinfo.cipf.es/) provides annotation of variants from next generation sequencing based on several different databases and repositories including dbSNP, 1000 Genomes Project, the GWAS catalog, OMIM, and COSMIC [36]. The provided annotations also include information on the regulatory or structural roles of the variants as well as the selective pressures on the affected genomic sites. Unlike other such tools, VARIANT utilizes a remote database and operates by interacting with this database through efficient RESTful Web Services. Currently VARIANT supports all human, mouse and rat genes. Analyzing variants generated by exome sequencing of families in which rare Mendelian diseases are segregated can be a time-consuming process.

### 6.11. VAR-MD

VAR-MD is a software tool to analyze variants derived from exome or whole genome sequencing in human pedigrees with Mendelian inheritance [51]. This algorithm outputs a ranked list of potential disease-causing variants based on predicted pathogenicity, Mendelian inheritance models, genotype quality, and population variant frequency data. This tool is unique in that it uses family-based annotation of sequence data to enhance mutation identification. VAR-MD is a Unix-based tool and is implemented in Python. Independent functions of the program are usually run sequentially. In order to facilitate parallel analysis of multiple data sets, VAR-MD utilizes Galaxy for distributed resource management.

The various variant annotation tools differ in the types of variants they process. All algorithms process SNPs and indels, but only a few, such as ANNOVAR and VAT, can handle SVs. These tools also differ in the computing environment in which they are implemented. Some rely on command-line operation while others operate using web-based interfaces or virtual machines in the cloud. Some tools utilize local databases while other use up-to-date remote databases. These various tools also differ in the genomic regions that they target. For example, SNPeffect focuses on the proteome while other tools focus on the less obvious, but still functionally relevant regions. From the long list of possible variants, through filtering and annotation, a smaller list of most probably causal variants is generated.

## 7. Future Outlook and Conclusion

While the current tools in all three stages of the bioinformatics analysis are adequate, more data will enable further significant improvements. New technology and algorithms may significantly shift the field in unforeseeable ways, but several future improvements are predictable as (1) sequencing reads increase in length, (2) more genomes are completed, and (3) annotation databases are better populated.

First, as sequencing technology increases the base pair read length, alignment will become more accurate. Shorter reads match with a greater number of genome sites. As reads grow in length, they can be mapped more precisely with fewer options and thus less room for error. This is especially true in regions with low complexity or a high number of repeats, classically very difficult regions to map. Longer reads will make alignment an easier problem.

Second, the process of variant calling will benefit from larger databases of completed genomes. A variant is derived from comparison to the reference genome, and our set of reference genomes continues to grow. This will enable variant calling based on ethnic background, or based on populations of genomes instead of a single reference genome or a small set of reference genomes.

Third, while filtering appears unlikely to change significantly, annotation and functional prediction will be improved by more data and more-populated databases. For filtering, since the genetic models and removal of normal variants from tumor variants are based only on the genetics and the samples under study, additional information from the databases will not change these aspects much. By contrast, the efficacy of annotation is directly related to what is present in known databases. Different dimensions of data, such as functional, pathway, biochemical, or genetic annotation can all be improved as more genomes are sequenced and annotated. Moreover, current predictive algorithms such as SIFT and Polyphen are dependent on current database annotation. If large numbers of human genomes are sequenced, analysis need not resort to merely predicting the effect of a single position; one can simply query that position in the millions of people that are sequenced and infer the deleterious effect.

Besides the more predictable changes that will follow naturally from more data, there are also opportunities for larger paradigm shifts in bioinformatics tools. First, emerging tools may be able to analyze samples not as a homogenous whole, but in ways that allow for tumor heterogeneity with differing populations of cells. Furthermore, single-cell and single-molecule methods are maturing. It is now more appreciated that the tumors consists of populations of cells, and that being able to determine the quantity and identity of these cells will not only help understand tumor population dynamics, but may also inform treatment and prognosis.

Second, thus far relatively few tools have integrated other high throughput modalities such as proteomics into genomic interpretation. In order to understand whether the mutation has biological significance, it is critical to know whether a gene is expressed on a transcript or protein level. As more multidimensional data is produced through projects such as ENCODE, TCGA, or 1000 Genomes, and high-throughput sample profiling becomes easier on a genomic, transcriptomic, and proteomic level, methods that can incorporate all this data will add power to the analysis.

Third, in additional to multidimensional data, there are also opportunities for systems biology methods to be incorporated to software packages. Protein-protein interaction datasets continue to grow as the human interactome is mapped, and knowledge of these molecular pathways can and should be integrated into genomics analysis. Understanding genes not only as isolated constructs but also as part of a greater system would better model the biological process.

Fourth, as more and more datasets are available and sequencing becomes cheaper, genomics analysis need no longer be based on a single genome, a comparison between an isolated pair of cancer genome samples, or larger, but still isolated, pedigrees. Current tools analyze single samples at a time and compare what is found with databases. Instead, tools that are able to analyze large numbers of genomes at the same time to sizes similar to genome-wide association studies will prove to be powerful.

Undoubtedly, the datasets used in genomics analysis will continue to grow in depth per individual and in the number of samples. Bioinformatics, more than ever before, will be the crucial step in making sense of the data flood. The incremental progress afforded by this flood will be critical and valuable, but researchers can also look forward to the yet-unknown paradigm shifts that loom over the horizon.

## Figures and Tables

**Table 1:** 

Program	Source type	Description	Website
Bowtie	Open source	Ungapped alignment	http://bowtie.cbcb.umd.edu/
		Refined use of FM Index using the BWT	
		Fast and memory-efficient alignment	
		Quality value output	

Bowtie2	Open source	Extends Bowtie approach to be useful for gapped alignment	http://bowtie-bio.sourceforge.net/bowtie2/index.shtml

SEAL	Open source	Comparison of alignment algorithms using simulated short read sequencing runs	http://compbio.case.edu/seal/

SOAP3	Open source	Gapped and ungapped alignment	http://www.cs.hku.hk/2bwt-tools/soap3/; http://soap.genomics.org.cn/soap3.html
		Specialized for detecting and genotyping SNPs	
		Hash table accelerates searching using BWT-based index	
		Reports multiple possibilities rather than single best match	
		Increased speed using GPU	

BWA, BWA-SW	Open source	Most common/standard method used	http://maq.sourceforge.net/
		Index with BWT that is faster than the hash-based index used for MAQ	
		Quality score reported	

mrFAST, mrsFAST	Open source	Seed-and-extend alignment method with hash table inex for reference genome	—
		Reports all read mappings instead of single best mapping Useful for CNVs, structural variants	

Novoalign	Commercially available	Novocraft's proprietary software with hashing strategy	http://www.novocraft.com/
		High accuracy for single end mapping	
		Focused on sensitivity	

SHRiMP/SHRiMP2	Open source	Specialized for SOLiD color-space reads using spaced seeds and SWA	http://compbio.cs.toronto.edu/shrimp/
		Also applicable for regular letter-space reads	
		Handles higher level of polymorphisms	

MAQ	Open source	Ungapped alignment	http://maq.sourceforget.net/
		Hash-based index with quality score for mapping	

Stampy	Open source	Hybrid mapping algorithm and statistical model, complementary to BWA	http://www.well.ox.ac.uk/project-stampy/

ELAND	Commercially available	Hash-based alignment program	http://www.illumina.com/

LAST aligner	Open source	Probablistic alignment quality scores as well as usual score matrix Useful for preprocessing for SNP/indel calling	http://last.cbrc.jp/

SARUMAN	Open Source	Mapping approach for single-end reads that returns all possible alignments with given error threshold with high speed using GPUs	http://www.cebitec.uni-bielefeld.de/brf/saruman/saruman.html
